# Early increase in circulating carbonic anhydrase IX during neoadjuvant treatment predicts favourable outcome in locally advanced rectal cancer

**DOI:** 10.1186/s12885-015-1557-6

**Published:** 2015-07-24

**Authors:** Helga Helseth Hektoen, Kjersti Flatmark, Yvonne Andersson, Svein Dueland, Kathrine Røe Redalen, Anne Hansen Ree

**Affiliations:** 1 Institute of Clinical Medicine, University of Oslo, P.O. Box 1171, Blindern, 0318 Oslo, Norway; 2 Department of Oncology, Akershus University Hospital, P.O. Box 1000, 1478 Lørenskog, Norway; 3 Department of Tumour Biology, Oslo University Hospital – Norwegian Radium Hospital, P.O.Box 4950, Nydalen, 0424 Oslo, Norway; 4 Department of Gastroenterological Surgery, Oslo University Hospital – Norwegian Radium Hospital, P.O.Box 4950, Nydalen, 0424 Oslo, Norway; 5 Department of Oncology, Oslo University Hospital – Norwegian Radium Hospital, P.O.Box 4950, Nydalen, 0424 Oslo, Norway

**Keywords:** Carbonic anhydrase IX, Chemotherapy, Metastasis, Radiotherapy, Rectal cancer, Survival, Tumour microenvironment

## Abstract

**Background:**

Locally advanced rectal cancer (LARC) comprises heterogeneous tumours with predominant hypoxic components. The hypoxia-inducible metabolic shift causes microenvironmental acidification generated by carbonic anhydrase IX (CAIX) and facilitates metastatic progression, the dominant cause of failure in LARC.

**Methods:**

Using a commercially available immunoassay, circulating CAIX was assessed in prospectively archived serial serum samples collected during combined-modality neoadjuvant treatment of LARC patients and correlated to histologic tumour response and progression-free survival (PFS).

**Results:**

Patients who from their individual baseline level displayed serum CAIX increase above a threshold of 224 pg/ml (with 96 % specificity and 39 % sensitivity) after completion of short-course neoadjuvant chemotherapy (NACT) prior to long-course chemoradiotherapy and definitive surgery had significantly better 5-year PFS (94 %) than patients with below-threshold post-NACT *versus* baseline alteration (PFS rate of 56 %; *p* < 0.01). This particular CAIX parameter, ΔNACT, was significantly correlated with histologic ypT0–2 and ypN0 outcome (*p* < 0.01) and remained an independent PFS predictor in multivariate analysis wherein it was entered as continuous variable (*p* = 0.04).

**Conclusions:**

Our results indicate that low ΔNACT, i.e., a weak increase in serum CAIX level following initial neoadjuvant treatment (in this case two cycles of the Nordic FLOX regimen), might be used as risk-adapted stratification to postoperative therapy or other modes of intensification of the combined-modality protocol in LARC.

**Trial registration:**

ClinicalTrials.gov NCT00278694

**Electronic supplementary material:**

The online version of this article (doi:10.1186/s12885-015-1557-6) contains supplementary material, which is available to authorized users.

## Background

Locally advanced rectal cancer (LARC) comprises heterogeneous tumours with hypoxic components, growing with locally advanced disease manifestations within the pelvic cavity. With contemporary treatment, commonly including fluoropyrimidine-based chemoradiotherapy (CRT) followed by resection of the residual tumour within its entire extension, local recurrence rates are low [1]. To the contrary, development of metastatic disease has remained the dominant cause of failure, typically reported to be 30–40 % of cases in recent clinical trials [2, 3]. Consequently, in the past decade, it has been increasingly appreciated that the sequence and combination of the various treatment modalities should be revamped. In October 2005, we launched a prospective non-randomised study composed of neoadjuvant oxaliplatin-containing chemotherapy (NACT; two cycles of the Nordic FLOX regimen) [4] before long-course CRT and radical surgery in order to intensify neoadjuvant LARC treatment, hypothesising such an approach might counteract the aggressive biology that evolves within the hostile microenvironment of hypoxic tumour components.

Hypoxia, causing metabolic changes towards enhanced glycolysis, is an important feature of the tumour microenvironment [5]. Additionally, the malignant phenotype promotes aerobic glycolysis as a consequence of diminished mitochondrial oxidative phosphorylation [6]. Tumours with such attributes rely on robust pH-regulating systems to combat the resulting excessive generation of lactic and carbonic acids [7]. Among these is the tumour-specific carbonic anhydrase IX (CAIX), a transmembrane enzyme that regulates extracellular and intracellular pH by catalysing the reversible hydration of carbon dioxide to bicarbonate and protons [8].

Accordingly, as demonstrated in a number of tumour types, CAIX over-expression is linked to poor prognosis. This observation has been attributed to the microenvironmental acidification that is generated by CAIX, causing breakdown of the extracellular matrix via proteinase activation and growth factor stimulation of cancer-associated fibroblasts, and consequently, augmentation of the metastatic potential [9, 10]. Moreover, the extracellular domain of the CAIX protein can be released from the cell surface by proteolytic cleavage [11]. In the present study, we asked whether CAIX, as retrieved in the circulation of LARC patients, might function as indicator of therapeutic outcome. Specifically, we employed prospectively archived serial serum samples collected during the neoadjuvant treatment course of our study patients, recognising the present follow-up time of five years for the vast majority of the cases, which would allow robust outcome analysis.

## Methods

### Patients and treatment

The study protocol was approved by the Institutional Review Board of the Norwegian Radium Hospital and the Regional Committee for Medical and Health Research Ethics, and was in accordance with the Helsinki Declaration. Written informed consent was required for participation. The patient population of 66 cases within the current analysis of serum CAIX was enrolled from October 5, 2005 through November 11, 2009. Patient eligibility criteria, evaluation procedures and review procedures of follow-up have been described in detail previously [12]. The treatment protocol consisted of two cycles of NACT (the Nordic FLOX regimen: oxaliplatin 85 mg/m^2^ on day 1 and daily bolus fluorouracil 500 mg/m^2^ and folinic acid 100 mg on days 1 and 2 every second week) followed by CRT. Radiation was delivered in daily 2-Gy fractions five days per week over a 5-week period. During the radiotherapy course, concomitant chemotherapy was given as oxaliplatin 50 mg/m^2^ once weekly and capecitabine 825 mg/m^2^ twice daily on days of radiotherapy. Surgery was planned 6–8 weeks after completion of the neoadjuvant treatment. In accordance with national guidelines, patients did not proceed to further treatment after surgery.

### Serum sampling

From the 66 study cases, serum had been collected at baseline (*n* = 66), at CRT commencement immediately following the two cycles of NACT (denoted post-NACT; *n* = 66), at CRT completion on the day of the 25th radiation fraction (denoted post-CRT; *n* = 54) and at evaluation of the neoadjuvant therapy four weeks after CRT completion (*n* = 50). The collection, processing and storage of samples followed a standardised protocol, where blood was drawn in plain serum tubes with no anti-coagulants for centrifugation to separate serum, which was left on ice for no more than one hour before storage at –80 °C. The analysis of serum CAIX was undertaken in June–July 2014 (i.e., after 55–105 months of storage).

### Assessment of serum CAIX

This analysis was undertaken with the Quantikine® Human CA9 Immunoassay (R&D Systems, Minneapolis, MN, USA) according to the manufacturer’s manual. Briefly, 100 μl of sample and standard control samples provided with the assay were incubated on microtiter plates that were pre-coated with a monoclonal antibody specific for CAIX. This was followed by incubation with an enzyme-linked polyclonal antibody against CAIX. A substrate solution containing a dye was added and colour intensity was measured by a microplate reader (Modulus™ Microplate Multimode Reader; Turner BioSystem, Sunnyvale, CA, USA). To correct for optical imperfections, plate readings at 540 nm were subtracted before the CAIX concentration was estimated from the standard curve and retrieved as pg/ml for each measurement. Assay performance was evaluated by plate-to-plate variation measures from patient samples within the high, medium and low range of CAIX levels, providing a coefficient of variation (CV) of 6.8 % in average for all of the three sample groups (specifically, CV = 5.7 % for the high level group, CV = 5.5 % for the medium level group and CV = 9.2 % for the low level group). Each of the individual serum samples was analysed in duplicate and the mean value was used in further calculations. For patients with paired sample measurements of post-NACT *versus* baseline (ΔNACT; *n* = 66) and post-CRT *versus* baseline (ΔCRT; *n* = 54), changes in the absolute value of serum CAIX following NACT alone or the combination of NACT and CRT were also calculated.

### Study endpoints

The resected tumour specimens were histologically evaluated for treatment response according to standard staging (ypTN). In this patient population of locally advanced tumours (mainly T3–4 cases), ypT0–2 outcome was considered as good response and correspondingly, ypT3–4 results were regarded as poor tumour shrinkage. Moreover, histologic tumour regression grade (TRG) was determined. In this, TRG1 represents absence of residual tumour cells (pathologic complete response), TRG2 corresponds to sparsely remaining tumour cells scattered throughout fibrosis (near-complete response), TRG3 shows residual tumour cells in fibrosis, TRG4 defines residual tumour cells outgrowing fibrosis and TRG5 refers to the absence of morphologic signs of treatment response (no fibrosis) [13]. Of note, when responding to neoadjuvant treatment, LARC frequently shows fragmentation into microscopic residual disease [14]. Consequently, it is rational to group TRG2 together with TRG1 as good histologic regression and correspondingly, the range of TRG3–5 scores as poor tumour cell response. Patients were scheduled for five years of follow-up after surgery. The clinical endpoint was progression-free survival (PFS). The present follow-up data was censored on August 8, 2013.

### Statistical analysis

All analyses were performed using IBM SPSS Statistics for Windows, Version 22.0 (IBM Corp., Armonk, NY, USA) or SigmaPlot 12.5 (Sysat Software Inc., Chicago, IL, USA). Continuous data were described with median and range and compared using non-parametric Mann-Whitney *U*-test, while categorical variables were described with proportions and percentages and compared using Pearson’s chi-square or Fisher’s exact tests. Diagnostic accuracy was assessed by receiver-operating characteristic (ROC) analysis. Estimated 5-year PFS was calculated from the time of study enrolment to the date of recurrent disease (diagnosis of local recurrence or distant metastasis), death of any cause or end of follow-up (five years after the date of surgery), whichever came first. Crude differences in survival were assessed using the Kaplan-Meier method and log-rank test. Associations between selected variables and PFS were modelled with univariate and multivariate Cox regression analysis. The results were expressed as hazard ratio with 95 % confidence interval. Variables that were statistically significant in univariate regression were entered into the multivariate model, but owing to limited statistical power, no more than three variables were included in the final model. The assumption of proportional hazards was tested by visual inspection of log-minus-log plots. All tests were two-sided. *p*-values less than 0.05 were considered statistically significant.

## Results

### Serum CAIX and clinical outcome

**Fig. 1:**
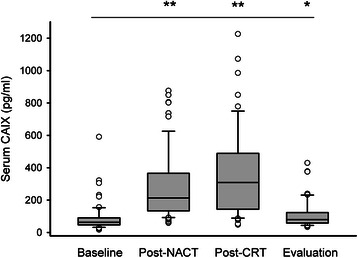
Serum carbonic anhydrase IX (CAIX) levels during neoadjuvant treatment of patients with locally advanced rectal cancer. Using a commercially available immunoassay, CAIX was measured in serum sampled from the study patients at baseline (*n* = 66), following four weeks of neoadjuvant chemotherapy (post-NACT; *n* = 66), at completion of a 5-week course of chemoradiotherapy (post-CRT; *n* = 54) and at evaluation of the neoadjuvant therapy four weeks later (*n* = 50). CAIX values are depicted by boxes (25th, 50th and 75th percentiles), bars (10th and 90th percentiles) and circles (outlier values). Distribution of CAIX values during the neoadjuvant course was different from baseline (* *p* < 0.05, ** *p* < 0.001; calculated by Mann-Whitney *U*-test)

Figure 1 illustrates the measured CAIX levels in serum samples from the patients that were present in the current study analysis. From a median baseline level of 63 pg/ml (range 17–591; *n* = 66), the median post-NACT value had increased to 213 pg/ml (range 59–875; *n* = 66) with further rise to 309 pg/ml (range 48–1226; *n* = 54) in the post-CRT samples. At evaluation, the median serum CAIX level had fallen to 80 pg/ml (range 34–429; *n* = 50). All group measurements within the neoadjuvant treatment course were significantly different from baseline.

Each individual CAIX dataset was divided in two groups above and below median value for further analysis (Additional file 1: Table S1). Statistical correlation was found between the clinical endpoint (PFS) and the absolute level of serum CAIX at NACT completion only and not for any of the other sampling points. But since both the post-NACT and post-CRT sampling points immediately followed the completion of a defined therapeutic modality, we further investigated whether the individual patient’s serum CAIX response (i.e., relative to baseline) at each of the sampling points might predict response to the combined-modality therapy in terms of disease outcome. Here, ΔNACT (median value 138 pg/ml, range from –4 to 659; *n* = 66) and ΔCRT (median value 208 pg/ml, range from –90 to 1022; *n* = 54) were assumed to reflect the change in serum CAIX following NACT alone or the entire treatment course of NACT and CRT, respectively. No correlation was found between ΔCRT and PFS.

**Fig. 2:**
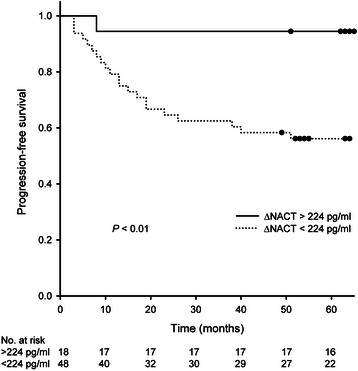
Serum carbonic anhydrase IX (CAIX) levels and progression-free survival in locally advanced rectal cancer. Progression-free survival was analysed (by the Kaplan-Meier method) for the population of 66 study patients with paired serum sample measurements of CAIX following neoadjuvant induction chemotherapy *versus* baseline (a variable termed ΔNACT), divided into two groups above (solid line) and below (dashed line) an estimated optimum cut-off ΔNACT value of 224 pg/ml. Difference between the two groups was significant (*p* < 0.01)

For PFS prediction, an optimum ΔNACT threshold of 224 pg/ml was found by ROC analysis (Additional file 2: Figure S1; area under curve 0.74, confidence interval 0.61–0.87; *p* < 0.01), yielding 96 % specificity, 39 % sensitivity, 94 % (17 of 18) positive predictive value and 44 % (21 of 48) negative predictive value. Estimated 5-year PFS was 94 % and 56 % when separating the patients above and below the ΔNACT threshold (*p* < 0.01) (Fig. 2).

### ΔNACT and tumour responses

Since ΔNACT values seemed to be a predictor of the resultant effect of the combined-modality treatment, further exploration of study endpoints was undertaken with this variable. Patient and tumour characteristics are given in Table 1. The two groups of patients with ΔNACT values above and below the estimated threshold were balanced (i.e., showed no statistical differences) with regard to baseline tumour characteristics.

**Table 1:** Study patients’ characteristics

		All patients	ΔNACT	ΔNACT	*p*-value
			<224 pg/ml	>224 pg/ml	
		(*n* = 66)	(*n* = 48)	(*n* = 18)	
		*n* (%)	*n* (%)	*n* (%)	
Median age (range), years		59 (30–73)	57 (30–72)	61 (50–73)	0.028^1^
Gender	Male	38 (58)	28 (58)	10 (56)	0.84^2^
	Female	28 (42)	20 (42)	8 (44)	
TN stage	T2–3	45 (68)	31 (65)	14 (78)	0.31^2^
	T4	21 (32)	17 (35)	4 (22)	
	N0–1	16 (24)	10 (21)	6 (33)	0.35^3^
	N2	49 (75)	37 (77)	12 (67)	
	ND	1	1	0	
Baseline CEA level	≤ULN	38 (58)	25 (52)	13 (72)	0.14^2^
	>ULN	28 (42)	23 (48)	5 (28)	
Baseline haemoglobin level	≥LLN	54 (82)	39 (81)	15 (83)	1^3^
	<LLN	12 (18)	9 (19)	3 (17)	
ypTN stage	ypT0–2	37 (56)	22 (46)	15 (83)	0.008^2^
	ypT3–4	28 (42)	25 (52)	3 (17)	
	ND	1	1	0	
	ypN0	46 (70)	29 (60)	17 (94)	0.009^2^
ypN	ypN1–2	19 (29)	18 (38)	1 (6)	
	ND	1	1	0	
TRG score	TRG1–2	44 (67)	30 (63)	14 (78)	0.28^2^
	TRG3–5	21 (32)	17 (35)	4 (22)	
	ND	1	1	0	

The population of 66 study patients with paired serum sample measurements of carbonic anhydrase IX following neoadjuvant induction chemotherapy *versus* baseline (a variable termed ΔNACT) was divided into two groups above and below an estimated optimum cut-off ΔNACT value of 224 pg/ml. Distribution of parameters between the two groups was compared using ^1^: Mann-Whitney *U*-test, ^2^_:_ Pearson’s chi-square test or ^3^: Fisher’s exact test

*ND* not determined (omitted from analysis), *CEA* carcinoembryonic antigen, *ULN* upper limit of normal, *LLN* lower limit of normal, *TRG* tumour regression grade

Significant correlation was found between ΔNACT and the histologic tumour stage following the neoadjuvant therapy, as patients with serum CAIX increase above 224 pg/ml had significantly higher rates of ypT0–2 and ypN0 outcome (*p* < 0.01). Of note, ΔNACT did not correlate with histologic TRG score. One of the 66 patients had disease progression in the pelvic cavity during the neoadjuvant treatment and therefore proceeded to palliative surgery. As a consequence, histologic tumour response data were missing and the single case was omitted from analysis.

### ΔNACT in PFS prediction

When last censored, the median follow-up time of this specific cohort of 66 patients was 63 months (range 3–65). Of these, 22 patients had experienced disease relapse; three had local recurrence and 19 had metastatic progression as the first event. As seen from Table 2, amongst baseline variables, young age and T4 stage were significantly associated with poorer PFS. In this univariate analysis, ΔNACT was entered as continuous data, and again the higher the value the better PFS. As expected, histologic treatment response was highly associated with clinical outcome, underpinned by the significant correlations of both ypT3–4 and ypN1–2 stages as well as TRG3–5 scores with adverse PFS. In multivariate analysis, into which only variables either present at baseline or during the neoadjuvant treatment were entered, the parameter requiring initiation of therapy (ΔNACT) was the only one that remained significantly associated with PFS (*p* = 0.04) (Additional file 3: Table S2).

**Table 2:** Progression-free survival in locally advanced rectal cancer

		HR^1^	95 % CI	*p*-value
ΔNACT		0.994	(0.990–0.999)	0.01
Age		0.953	(0.914–0.993)	0.02
TN stage	T2–3			
	T4	2.71	(1.17–6.27)	0.02
	N0–1			
	N2	1.22	(0.449–3.30)	0.70
Baseline CEA level	≤ULN			
	>ULN	1.85	(0.799–4.29)	0.15
ypTN	ypT0–2			
	ypT3–4	6.10	(2.22–16.7)	<0.001
	ypN0			
	ypN1–2	2.84	(1.20–6.70)	0.02
TRG score	TRG1–2			
	TRG3–5	3.00	(1.27–7.08)	0.01

Adjusted hazard ratio (HR) with 95 % confidence interval (CI) was calculated by univariate Cox regression analysis for each of the indicated variables

*ΔNACT* paired serum sample measurements of carbonic anhydrase IX following neoadjuvant induction chemotherapy *versus* baseline, *CEA* carcinoembryonic antigen, *ULN* upper limit of normal, *TRG* tumour regression grade

^1^: HR less than 1 indicates that patients had higher probability of favourable progression-free survival

## Discussion

The present study of circulating CAIX response during neoadjuvant treatment of LARC presented novel findings. Patients who from their individual baseline level showed strong increase in serum CAIX after completion of a relatively short course of NACT prior to long-course CRT had significantly better PFS than patients with low post-NACT *versus* baseline alteration. This particular CAIX parameter, ΔNACT, remained the only independent PFS predictor in multivariate analysis of variables presenting either at baseline or during the neoadjuvant treatment. Strikingly, high ΔNACT was significantly correlated with ypT0–2 and ypN0 outcomes after the full neoadjuvant treatment. In contrast, ΔNACT and histologic TRG score of the surgical specimen were unrelated parameters. Hence, in this population of mainly T3–4 rectal cancer patients, a strong serum CAIX response to initial treatment reflected forthcoming tumour down-staging (ypT0–2) and node sterilisation (ypN0), but not necessarily tumour cell death (TRG score), and ultimately a favourable PFS.

In this study, the number of cases from whom serum samples were available was rather small (ranging from 66 at baseline to 50 before surgery) with only 22 patients had reported a PFS event. Consequently, the value of multivariate regression analysis was limited. The study findings should ideally have been evaluated in an independent patient cohort where cases had undergone combined-modality radiotherapy with curative intent for a locally advanced malignancy along the simultaneous prospective serial sampling of serum (or plasma) throughout the treatment course and where, additionally, short-term (tumour response) and long-term (PFS or an equivalent main event) outcome data were available. Our attempts to identify such a material have not been successful.

In rectal cancer, a limited number of studies have reported CAIX expression analysed by immunohistochemistry in surgical specimens from patients undergoing primary surgery [15–17] or in baseline biopsy samples or surgical specimens from patients given neoadjuvant therapy [16, 18, 19]. Interestingly, patients who obtained weak CAIX staining in surgical specimens after neoadjuvant short-course (radiation) or long-course treatment had significantly better disease-specific survival than patients with resulting strong CAIX tumour expression [16]. The authors also divided the long-course group into patients that had been given CRT or radiation only and found significantly higher percentage of high CAIX expression in surgical samples from patients that had not received concomitant chemotherapy; of note, the radiotherapy only group consisted of only eight cases. Still, it was contended that chemotherapy, which in this case was fluoropyrimidine-based regimens, might improve tumour oxygenation and the final patient outcome [16].

To our knowledge, the present study is the first to report on the profile of circulating CAIX during neoadjuvant treatment in LARC. Conceptually, serum CAIX levels at the post-NACT and post-CRT sampling points reflected the contribution of either NACT or NACT followed by CRT within the entire combined-modality protocol. To our initial surprise, ΔNACT but not ΔCRT appeared to be a predicting factor of PFS. Hence, the impact of two cycles of Nordic FLOX but not full treatment with NACT and CRT on tumour release of CAIX into the circulation seemed to be critical for the ultimate outcome. Patients with increase in serum CAIX of more than 224 pg/ml from the baseline level were more likely to obtain tumour down-staging and node sterilisation, which is recognised to translate into long-term survival benefits [20]. It is further noteworthy that the TRG outcome, which represents the degree of surviving tumour cells relative to fibrosis (i.e., histologically responding tumour components) in the surgical specimen, was statistically unrelated to ΔNACT.

A reasonable interpretation of the two observations might be that a response of CAIX-expressing stromal cells to the initial therapy would be conditional for an excellent PFS (in this case, an estimated 5-year rate of 94 %). In this context, an interesting report published by pathologists looked at tumour CAIX expression in more than one hundred patients who had undergone primary surgery for colorectal carcinoma [15]. Intriguingly, CAIX expression was demonstrated both in tumour epithelial cells (78 % of cases) and tumour-associated stromal cells (37 % of cases), and half of cases that lacked epithelial CAIX expression were positive for stromal staining. Of particular note, whereas epithelial CAIX expression had no impact on survival outcome, stromal positive patients had significantly poorer overall survival, and this remained an independent prognostic factor in multivariate analysis. The role of CAIX-expressing stromal cells on clinical outcome has also been highlighted in breast, lung and head-and-neck carcinoma [21–23].

With reference to interpretation of findings mentioned above [16] as well as to the data from the present study, it is tempting to speculate about the mechanistic role of chemotherapy in shedding of the CAIX protein. In our study, induction chemotherapy in terms of two cycles of the Nordic FLOX regimen was introduced in order to intensify the multimodal treatment protocol, hypothesising such an approach might counteract the aggressive biology of a hypoxic tumour microenvironment with the ultimate aim of improving long-term survival. The microenvironmental acidification generated by CAIX induces metalloproteinase activation [10], which next causes proteolytic CAIX cleavage and consequently its ectodomain shedding [11]. Furthermore, experimental data supports the notion that chemotherapy may enhance such shedding [11]. One might therefore speculate that chemotherapy specifically targets hypoxic, CAIX-expressing tumour components. However, it remains unclear whether the ΔNACT response observed in the present study was contingent on the Nordic FLOX regimen as such or whether an early CAIX response, irrespective of the therapeutic modality, would have correlated with a favorable long-term outcome. In the forthcoming, concerns and implications for excisting clinical practice will be discussed.

Despite contemporary multimodal therapy, a substantial number of rectal cancer patients will experience metastatic dissemination. None of the recently published randomised trials on combined-modality treatment of LARC has demonstrated survival benefit [24]. Like our study, one strategy that has been argued to improve cure rate would be to introduce systemic therapy within the neoadjuvant treatment course to eradicate occult micrometastases that might already exist at the presentation of the local disease. Induction chemotherapy involving oxaliplatin has been included in several previous neoadjuvant LARC trials [25–31]. However, it has been a concern that sequential treatment causes protraction of the total treatment time, which is undesirable in terms of delaying commencement of radiation as a well-documented modality to eliminate the source of clonogenic tumour cells with metastatic potential. Additionally, in this context, the administration of NACT might influence patient compliance with subsequent CRT and the efficacy of CRT, in particular the possibility that NACT might select for radioresistant tumour clones. To comply with these concerns, our strategy was to administer NACT limited to only two cycles of the Nordic FLOX regimen before CRT and definitive surgery.

Our subsequent CRT regimen involved concomitant oxaliplatin and the fluoropyrimidine analogue capecitabine. The role of oxaliplatin as an additional component of CRT in LARC is controversial. During the conduct of our trial, emerging data from three different randomised studies suggested no additional clinical benefit but significantly enhanced acute toxicity of adding oxaliplatin to fluoropyrimidine-based CRT [2, 32–34], whereas a fourth study demonstrated significantly improved rate of histologic complete response in the oxaliplatin-supplemented group [35].

Within this frame of reference, it is noteworthy that a strong serum CAIX response to the two FLOX cycles translated into an advantageous PFS. Assuming that CAIX is a tumour-specific protein that is released into the circulation as a direct response to therapy [11], i.e., that no other tissue of origin contributes as a toxic response to the given therapy, the absolute change in serum CAIX level might be utilised in risk-adapted individualisation of LARC treatment. Currently, the use of postoperative adjuvant chemotherapy, as being given in colon cancer, has been adopted at a number of centres internationally despite the lack of evidence-based research on which to base treatment decisions [1, 36]. In most Nordic countries, postoperative treatment of rectal cancer is offered only on specific, individual-based indications. Circulating CAIX response to the initial therapy, as biomarker for systemic risk assessment, might assist in stratification of refractory cases to postoperative treatment, which might be conventional chemotherapy or targeted therapeutics directed towards hypoxic tumour mechanisms.

## Conclusion

Our results suggest that a strong serum CAIX response to initial neoadjuvant treatment in LARC reflects forthcoming tumour down-staging and node sterilisation and might be used as risk-adapted stratification to adjusted multimodal treatment protocols.

## Additional files

Additional file 1: Table S1. Serum carbonic anhydrase IX (CAIX) variables and progression-free survival in locally advanced rectal cancer. Each of six datasets of serum CAIX measurements in study patients was divided into two groups above and below the individual median value. Differences between the two groups were calculated by log-rank test. (PDF 74 kb)
Additional file 2: Figure S1. Receiver-operating characteristic analysis of the treatment-induced changes in serum carbonic anhydrase IX (CAIX) level for the prediction of progression-free survival in locally advanced rectal cancer. In 66 study patients with paired serum CAIX measurements following two cycles of neoadjuvant chemotherapy *versus* baseline (a variable termed ΔNACT), diagnostic accuracy for prediction of 5-year progression-free survival had 96 % specificity and 39 % sensitivity at the ΔNACT cut-off value of 224 pg/ml. (PDF 94 kb)
Additional file 3: Table S2. Progression-free survival in locally advanced rectal cancer. Adjusted hazard ratio with 95 % confidence interval was calculated by multivariate Cox regression analysis for each of the indicated variables. (PDF 72 kb)

## Abbreviations

CAIX: Carbonic anhydrase IX
CRT: Chemoradiotherapy
CV: Coefficient of variation
LARC: Locally advanced rectal cancer
NACT: Neoadjuvant chemotherapy
PFS: Progression-free survival
ROC: Receiver-operating characteristic
TRG: Tumour regression grade
