# Supplementary material for: Early increase in circulating carbonic anhydrase IX during neoadjuvant treatment predicts favourable outcome in locally advanced rectal cancer
Source: BMC Cancer. 2015 Jul 24;15:543. doi: 10.1186/s12885-015-1557-6 (PMC4513373; doi:10.1186/s12885-015-1557-6)
Supplement: Additional file 1: Table S1. — Serum carbonic anhydrase IX (CAIX) variables and progression-free survival in locally advanced rectal cancer. Each of six datasets of serum CAIX measurements in study patients was divided into two groups above and below the individual median value. Differences between the two groups were calculated by log-rank test. (PDF 74 kb) [file 12885_2015_1557_MOESM1_ESM.pdf]

Additional file 1: Table S1. Serum carbonic anhydrase IX (CAIX) variables and progression-free survival (PFS) in locally advanced rectal cancer. Each of six datasets of serum CAIX measurements in study patients was divided into two groups above and below the individual median value. Differences between the two groups were calculated by log-rank test.

	**Median CAIX value (range), pg/ml**	***n***	**Difference in PFS, *p*-value**
Baseline	63 (17–591)	66	0.60
Post-NACT	213 (59–875)	66	0.03
Post-CRT	309 (48–1226)	54	0.45
Evaluation	80 (34–429)	50	0.75
ΔNACT	138 (from –4 to 659)	66	0.01
ΔCRT	208 (from –90 to 1022)	54	0.09

NACT: neoadjuvant chemotherapy.
CRT: chemoradiotherapy.
ΔNACT: paired serum sample measurements of CAIX following NACT *versus* baseline.
ΔCRT: paired serum sample measurements of CAIX following NACT and CRT *versus* baseline.
