# Supplementary material for: Early increase in circulating carbonic anhydrase IX during neoadjuvant treatment predicts favourable outcome in locally advanced rectal cancer
Source: BMC Cancer. 2015 Jul 24;15:543. doi: 10.1186/s12885-015-1557-6 (PMC4513373; doi:10.1186/s12885-015-1557-6)
Supplement: Additional file 2: Figure S1. — Receiver-operating characteristic analysis of the treatment-induced changes in serum carbonic anhydrase IX (CAIX) level for the prediction of progression-free survival in locally advanced rectal cancer. In 66 study patients with paired serum CAIX measurements following two cycles of neoadjuvant chemotherapy versus baseline (a variable termed ΔNACT), diagnostic accuracy for prediction of 5-year progression-free survival had 96 % specificity and 39 % sensitivity at the ΔNACT cut-off value of 224 pg/ml. (PDF 94 kb) [file 12885_2015_1557_MOESM2_ESM.pdf]

Additional file 2: Figure S1. Receiver-operating characteristic analysis of the treatment-induced changes in serum carbonic anhydrase IX (CAIX) level for the prediction of progression-free survival in locally advanced rectal cancer.

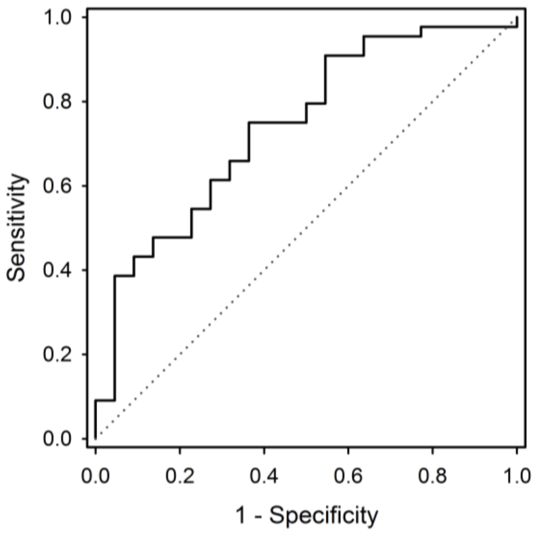

In 66 study patients with paired serum CAIX measurements following two cycles of neoadjuvant chemotherapy *versus* baseline (a variable termed ΔNACT), diagnostic accuracy for prediction of 5-year progression-free survival had 96% specificity and 39% sensitivity at the ΔNACT cut-off value of 224 pg/ml.
