# Supplementary material for: Early increase in circulating carbonic anhydrase IX during neoadjuvant treatment predicts favourable outcome in locally advanced rectal cancer
Source: BMC Cancer. 2015 Jul 24;15:543. doi: 10.1186/s12885-015-1557-6 (PMC4513373; doi:10.1186/s12885-015-1557-6)
Supplement: Additional file 3: Table S2. — Progression-free survival in locally advanced rectal cancer. Adjusted hazard ratio with 95 % confidence interval was calculated by multivariate Cox regression analysis for each of the indicated variables. (PDF 72 kb) [file 12885_2015_1557_MOESM3_ESM.pdf]

Additional file 3: Table S2. Progression-free survival in locally advanced rectal cancer. Adjusted hazard ratio (HR) with 95% confidence interval (CI) was calculated by multivariate Cox regression analysis for each of the indicated variables.

		**HR[1]**	**95% CI**	***p*-value**
ΔNACT		0.995	0.990–1.00	0.04
Age		0.968	0.926–1.01	0.14
T stage	T2–3			
	T4	1.78	0.722–4.41	0.20

ΔNACT: paired serum sample measurements of CAIX following two cycles of neoadjuvant chemotherapy *versus* baseline.
[1]: HR less than 1 indicates that patients had higher probability of favourable progression-free survival.
